# Moss cell walls: structure and biosynthesis

**DOI:** 10.3389/fpls.2012.00166

**Published:** 2012-07-19

**Authors:** Alison W. Roberts, Eric M. Roberts, Candace H. Haigler

**Affiliations:** ^1^Department of Biological Sciences, University of Rhode Island,Kingston, RI, USA; ^2^Department of Biology, Rhodes Island College,Providence, RI, USA; ^3^Department of Crop Science, North Carolina State University,Raleigh, NC, USA; ^4^Department of Plant Biology, North Carolina State University,Raleigh, NC, USA

**Keywords:** cell wall, polysaccharide, cellulose, cellulose synthesis complex, glycosyl transferase, moss, *Physcomitrella patens*

## Abstract

The genome sequence of the moss *Physcomitrella patens* has stimulated new research examining the cell wall polysaccharides of mosses and the glycosyl transferases that synthesize them as a means to understand fundamental processes of cell wall biosynthesis and plant cell wall evolution. The cell walls of mosses and vascular plants are composed of the same classes of polysaccharides, but with differences in side chain composition and structure. Similarly, the genomes of *P. patens* and angiosperms encode the same families of cell wall glycosyl transferases, yet, in many cases these families have diversified independently in each lineage. Our understanding of land plant evolution could be enhanced by more complete knowledge of the relationships among glycosyl transferase functional diversification, cell wall structural and biochemical specialization, and the roles of cell walls in plant adaptation. As a foundation for these studies, we review the features of *P. patens* as an experimental system, analyses of cell wall composition in various moss species, recent studies that elucidate the structure and biosynthesis of cell wall polysaccharides in *P. patens*, and phylogenetic analysis of *P. patens* genes potentially involved in cell wall biosynthesis.

## MOSS BIOLOGY AND EVOLUTION

The common ancestor of land plants is believed to have resembled extant mosses in having a biphasic life cycle with a dominant haploid gametophyte and rudimentary adaptations for tolerating the aerial environment. Because they have retained these characteristics, mosses are often referred to as “lower” plants (Mishler and Oliver, 2009). Despite this designation, mosses are highly successful, comprising more than 10,000 species adapted to diverse habitats ranging from submerged aquatic to desert (Buck and Goffinet, 2000). Mosses differ from vascular plants in the strategy they employ to survive in the dry aerial environment. Vascular plants are homeohydric with a thick cuticle to reduce dehydration, roots to extract water from the soil, and vascular tissue to distribute water internally. In contrast, mosses are poikilohydric, depending on a surface film of free water to maintain hydration. Although some mosses are confined to aquatic habitats, many are dehydration tolerant and some are desiccation tolerant (Mishler and Oliver, 2009).

**FIGURE 1:**
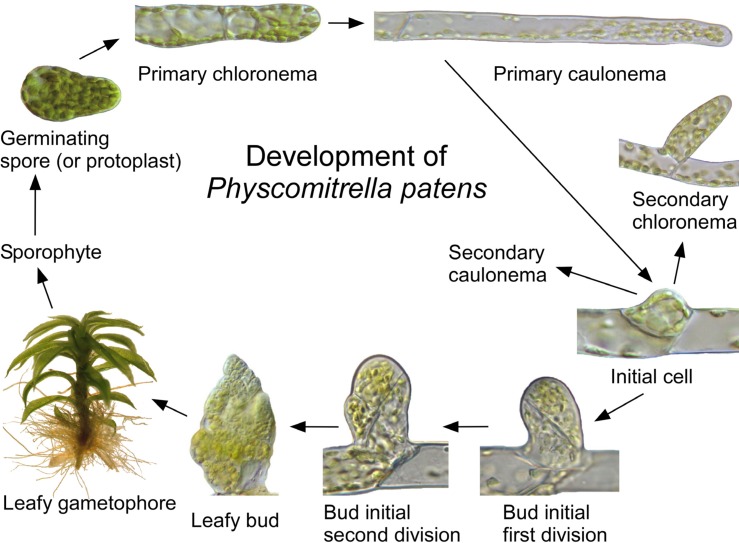
**Development of *Physcomitrella patens***. A haploid spore or protoplast germinates to form a primary chloronema with numerous chloroplasts and transverse cross walls, which subsequently differentiates into a more rapidly growing primary caulonema with fewer chloroplasts and oblique cross walls. The caulonemal subapical cells divide to produce initials cells, which develop into lateral secondary chloronemata, secondary caulonemata (not shown), or buds. Buds develop into leafy gametophores, which produce apical gametangia. Fertilization of an egg by swimming sperm at the gametophore apex produces a zygote, which develops into a diploid sporophyte consisting of a stalk and sporangium (not shown). Meiotic divisions within the sporangium generate haploid spores.

The life cycle, morphology, and biochemistry of mosses have been influenced by selective pressure associated with poikilohydry. Because cellular water status is controlled by surface absorption and diffusion, moss organs are small and thin, and lignin is not required to provide support against gravity or the negative pressures generated during transpiration (Mishler and Oliver, 2009). In most mosses, haploid spores produce protonemal filaments that extend by apical division and tip growth (**Figure 1**). The filaments produce buds that develop into leafy gametophores, which enlarge by diffuse growth. Haploid spores are produced by diploid sporophytes, which develop from eggs fertilized by swimming sperm at the gametophore apex (Schumaker and Dietrich, 1998).

Although structurally simple, moss gametophytes contain different cell types. The protonema is differentiated into chloroplast-rich chloronemal cells and caulonemal cells, which elongate three times faster (Menand et al., 2007). In response to dehydration stress, protonemal cells resume division and differentiate into thick-walled, dehydration-tolerant brachycytes, and subtending tmema cells, which undergo programmed cell death (Decker et al., 2006). Gametophores include a stem, leaves, axillary hairs, and rhizoids. The stem typically consists of small, thick-walled epidermal and subepidermal cells, thin-walled parenchyma cells, and conducting cells. The conducting cells include hydroids and leptoids, which are functionally analogous to xylem and phloem (Buck and Goffinet, 2000). Similar to tracheary elements, hydroids are dead at maturity and connected by perforations, but they lack thick lignified secondary cell walls (Hebant, 1977). Leaves are typically one cell layer thick except for the midribs and margins, which may consist of multiple layers of differentiated cells. Leaf cells of *Sphagnum* species include photosynthetic chlorocytes and hyalocytes with elaborate cell wall thickenings. Other leaf cell specializations include papillae and various surface elaborations. Additional differentiated cell types form the gametangia, gametes, and sterile paraphyses. The sporophyte stalk, sporangium, and spores also consist of specialized cell types (Buck and Goffinet, 2000), including stomata (Sack and Paolillo, 1983).

Although mosses share a poikilohydric ecological strategy and common body plan, their diversification and colonization of different habitats have been accompanied by the evolution of specialized morphological and biochemical adaptations that must be considered when inferring evolutionary trends from comparative studies of mosses and vascular plants. The mosses diverged from the land plant lineage between the liverworts and the hornworts, which most recent phylogenies place as sister to the vascular plants. The moss lineage includes the “true mosses” and three early divergent and ecologically specialized lineages, the aquatic Sphagnales, the desiccation-tolerant rock-dwelling Andreales, and the morphologically diverse Polytricales (Mishler and Oliver, 2009). Whereas mosses have retained primitive aspects of cell wall structure and composition due to poikilohydry, they have also evolved special cell wall adaptations that enabled them to colonize diverse habitats.

## *PHYSCOMITRELLA PATENS*, THE MODEL MOSS SPECIES

As a member of the Funariales, *Physcomitrella patens* occupies a phylogenetic position at the base of the true mosses. As an inhabitant of moist soils that tolerates dehydration, but not desiccation, it represents a “primitive moss ecology” (Mishler and Oliver, 2009). This lack of specialization for extreme conditions combined with abundant genomic resources (Rensing et al., 2008), efficient production of transgenic genotypes, and ease of culture and experimental manipulation (Cove, 2005) provides an opportunity to relate the diversification of gene families to innovations in cell wall composition, structure, and development that accompanied the adaptation of plants to life on land. Other advantages of *P. patens* include the ability to produce large amounts of tissue consisting of a single cell type (chloronemal filaments) and rapid cell wall regeneration in protoplasts (Lee et al., 2005a; Lawton and Saidasan, 2011; Roberts et al., 2011).

## CELL WALL ANALYSIS

Cell wall polysaccharide composition has been investigated in several moss species, including *P. patens*. As a complement to biochemical methods, immunological and affinity approaches employing antibodies and carbohydrate binding modules that recognize a variety of cell wall polysaccharides (Knox, 2008) have been used to examine the distribution of polysaccharides and with the microarray method known as comprehensive microarray polymer profiling (CoMPP; Moller et al., 2007).

### CELLULOSE

**FIGURE 2:**
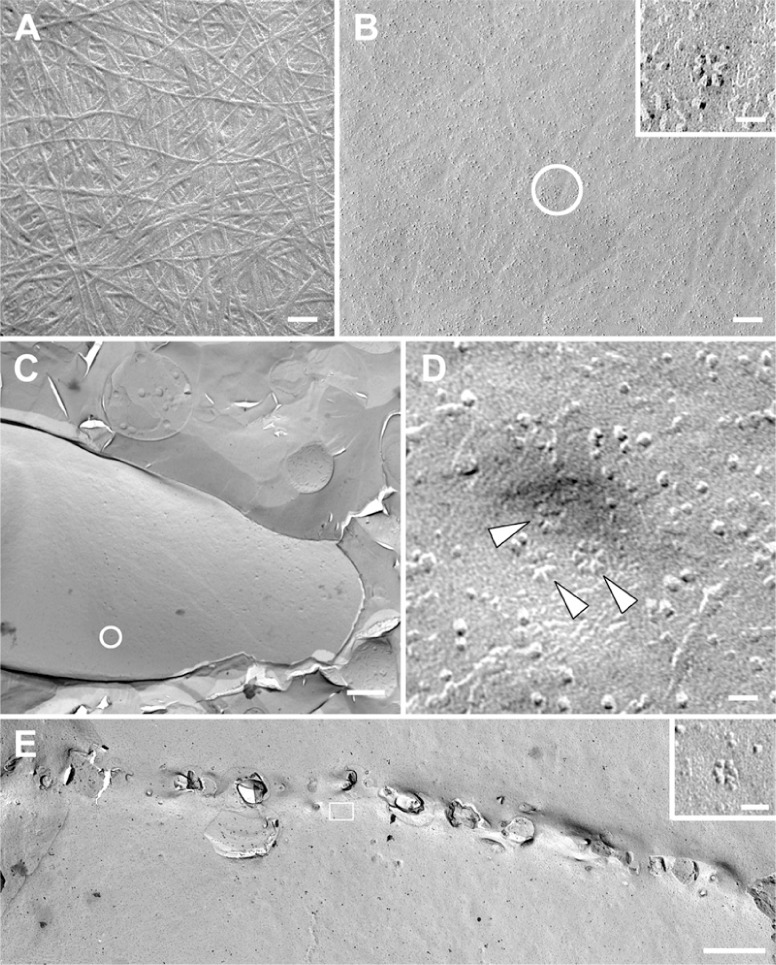
**Transmission electron micrographs of extracted and shadowed cell walls and platinum–carbon freeze fracture replicas from *Physcomitrella patens* protonemal filaments**. **(A)** Cell wall extracted with 1 N NaOH and shadowed with platinum–carbon, showing microfibrils (bar = 100 nm). **(B)** Plasma membrane fracture with microfibril impressions and rosette-type cellulose synthesis complexes (circle and inset, bars = 100 nm for **(B)**, 20 nm for inset). **(C)** Plasma membrane fracture of a protonemal tip (bar = 2 μ). **(D)** Higher magnification view of the circled area in **D**, showing a presumed secretory vesicle containing at least one rosette-type cellulose synthesis complex (top arrowhead). The other two rosette cellulose synthesis complexes (bottom arrowheads) may have been in the plasma membrane beforehand or in the midst of secretion from the same vesicle when the cell was frozen (bar = 20 nm). **(E)** Fusion of a cell plate with the plasma membrane in a dividing protonemal tip cell with associated rosette cellulose synthesis complexes (box and inset, bars = 1 μ for **(E)**, 20 nm for inset).

Cellulose exists in the cell walls of all mosses that have been examined. It was detected in *P. patens* by CoMPP, sugar linkage analysis, and staining with Tinopal and CBM3A, a probe specific for crystalline cellulose (Kremer et al., 2004; Lee et al., 2005b, 2011; Moller et al., 2007; Nothnagel and Nothnagel, 2007; Goss et al., 2012). As is typical for cellulose, 5–20 nm wide microfibrils are visible in extracted and shadowed cell walls (**Figure 2A**). Microfibril impressions also occur in freeze-fractured plasma membranes (**Figure 2B**). Fibrils detected by atomic force microscopy on the surface of air-dried protonemal filaments were 250 nm in diameter (Wyatt et al., 2008), which is consistent with cellulose aggregation upon drying.

The *P. patens* genome includes seven *Cellulose Synthase* genes (*PpCESA3*,-*4*,-*5*,-*6*,-*7*,-*8*, and -*10* = -*11*) and three *CESA* pseudogenes (*PpCESA1*,-*2*, and -*9*; Roberts and Bushoven, 2007; Yin et al., 2009; Wise et al., 2011). Whereas seed plant CESAs are specialized for primary and secondary cell wall deposition, the CESAs of *P. patens* may be specialized for tip and diffuse growth. *PpCESA5* is required for leafy gametophore morphogenesis and is upregulated by cytokinin, which also induces gametophore development (Goss et al., 2012). Based on over-representation in EST libraries from cytokinin-treated cultures, *PpCesA4* and -*10* may also be involved in gametophore development. In contrast, *PpCESA6* is expressed in tip-growing protonemal filaments, rhizoids, and axillary hairs (Wise et al., 2011). Knockout mutants of *PpCESA6* and -*7*, which differ by three amino acids, have no morphological phenotype. Shorter gametophores were observed in one *PpCESA6*/*7* double mutant line (Wise et al., 2011). Analysis of additional *PpCESA* knockout mutants will be required to fully understand CESA diversification and functional specialization in *P. patens*.

Mosses including *Funaria hygrometrica* (Reiss et al., 1984; Rudolph and Schnepf, 1988; Rudolph et al., 1989) and *P. patens* have rosette-type cellulose synthesis complexes (CSCs). CSCs are abundant near apical cell tips, some apparently emerging from secretory vesicles (**Figures 2C,D**), and adjacent to forming cell plates (**Figure 2E**). Rosette-type CSCs of seed plants contain three CESA isoforms, and it has been suggested that *CESA* diversification was a prerequisite for rosette CSC evolution (Doblin et al., 2002). However, phylogenetic analyses indicate that the *PpCESA*s are not orthologs of the functionally specialized seed plant *CESA*s and that the common ancestor of mosses and seed plants had a single *CESA*. Thus, the hetero-oligomeric CSCs of seed plants evolved from homo-oligomeric rosettes, which may still exist in *P. patens* (Roberts and Bushoven, 2007). Yet, *P. patens* could have hetero-oligomeric CSCs if they evolved independently in mosses and seed plants. Published CESA phylogenies indicate that the divergence that produced primary and secondary cell wall CESAs preceded the diversification that resulted in hetero-oligomeric CSCs (Tanaka et al., 2003; Djerbi et al., 2005; Nairn and Haselkorn, 2005; Ranik and Myburg, 2006; Roberts and Bushoven, 2007; Kumar et al., 2009; Carroll and Specht, 2011). This implies that hetero-oligomeric CSCs evolved independently from homo-oligomeric primary and secondary CSCs. Although this scenario seems unparsimonious, the theory of constructive neutral evolution recently demonstrated for the V-ATPase complex in yeast (Doolittle, 2012; Finnigan et al., 2012) postulates that multisubunit complexes, such as CSCs, are driven towards a hetero-oligomeric state. Like CSCs, the transmembrane ring of yeast V-ATPase consists of three paralogous, but non-interchangeable, protein subunits. By reconstructing the common ancestor of two of these subunits and reintroducing historical mutations, Finnigan et al. (2012) showed that a gene duplication followed by complementary loss of specific interfaces involved in protein–protein interactions was responsible for the evolution of subunits that differ only in the positions that they occupy within the complex. In this process, a high-probability loss-of-function (i.e., the inability to interact with like subunits) is initially independent of selection, but the hetero-oligomeric condition becomes locked-in by selection as mutations accumulate. This driving of multimeric protein complexes toward increased complexity explains how the hetero-oligomeric state could have evolved independently in primary and secondary cell walls CSCs in seed plants and, possibly, in *P. patens*.

### CROSS-LINKING GLYCANS

Xyloglucan has been detected in various moss species by the presence of isoprimeverose in driselase digests (Popper and Fry, 2003) and in *P. patens* by CoMPP using antibodies directed against non-fucosylated xyloglucan (Moller et al., 2007). Detailed structural analysis confirmed the absence of fucopyranosyl residues and revealed that *P. patens* xyloglucan has an XXGGG branching pattern and novel branched side chains containing galactosyluronic acid and arabinopyranosyl residues (Peña et al., 2008). Based on immunolabeling the leaves and stems are enriched in xyloglucan (Kulkarni et al., 2012). The five members of the *P. patens* *CSLC* family of putative xyloglucan synthases (Cocuron et al., 2007) form a clade separate from seed plant *CSLC*s (Roberts and Bushoven, 2007). The *P. patens* genome also encodes putative homologs of the *Arabidopsis* xyloglucan xylosyl transferases XXT1 and XXT2, and galactosyl transferases MUR3 and GT18 (Peña et al., 2008). Because xylopyranosyl residues of *P. patens* xyloglucan are substituted with galactosyluronic acid or arabinopyranosyl residues instead of galactopyranosyl residues, it was suggested that the *P. patens* homologs differ from MUR3/GT18 in substrate specificity (Peña et al., 2008). Although the *P. patens* genome encodes several members of GT37, which includes the *Arabidopsis* xyloglucan fucosyl transferase FUT1, sequence similarity is low (Peña et al., 2008) and the *P. patens* sequences are not in the FUT1 subclade (Del Bem and Vincentz, 2010), consistent with the lack of xyloglucan fucosylation. Differences in side chain frequency in xyloglucans extracted from protonemal and gametophore cell walls may be related to the roles of xyloglucan in tip and diffuse growth (Peña et al., 2008).

Mannans are present in walls of various bryophytes based on chemical analysis (Geddes and Wilkie, 1971; Popper and Fry, 2003) and in *P. patens* based on CoMPP, chemical analysis, and immunolabeling (Liepman et al., 2007; Moller et al., 2007; Nothnagel and Nothnagel, 2007; Lee et al., 2011). The *P. patens* genome includes three *CSLA* genes (Roberts and Bushoven, 2007), at least two of which have mannan/glucomannan synthase activity when expressed in insect cells (Liepman et al., 2007).

A recent immunolabeling study failed to detect xylan-specific epitopes in eight moss species, including *F. hygrometrica* (Carafa et al., 2005). However, xylan-specific epitopes were detected in *P. patens* by CoMPP, and sugar linkage analysis confirmed the presence of β-1,4-linked xylan (Moller et al., 2007). A detailed structural analysis revealed glucuronoxylan with a 1,4-linked β-D-xylan backbone and α-D-glucosyluronic acid side chains, but no 4-*O*-methyl-α-D-glucosyluronic acid side chains, indicating that *O*-methylation of glucosyluronic acid evolved after divergence of mosses and vascular plants. The *P. patens* xylan is also unusual in having pairs of side chains separated by a single xylosyl residue and possibly an unidentified pentosyl residue. Xylans from both seedless vascular plants and *P. patens* lack the reducing-end sequence characteristic of seed plant xylans (Kulkarni et al., 2012). Xylan has been immunolocalized in leaf cells and axillary hairs in *P. patens*, but little or none was detected in the stems and protonemal filaments (Kulkarni et al., 2012).****Xylan backbone synthesis in *Arabidopsis* involves members of GT43 (IRX-9, IRX-9L, IRX-14, IRX-14L) and GT47 (IRX-10, IRX-10L). The major clades containing these proteins and their putative seed plant orthologs include *P. patens* sequences (Kulkarni et al., 2012). However, the *P. patens* members of the IRX-9/IRX-9L clade may not be their direct orthologs. Biosynthesis of the xylan reducing-end sequence in *Arabidopsis* involves members of GT8 (IRX-8/GAUT-12, PARVUS/GATL-1) and GT47 (IRX7). The *P. patens* genome encodes three basal members of the subclade containing GAUT-12 and three other AtGAUTs, five basal members of the clade containing GATL1 (Yin et al., 2010), and three basal members of the subclade containing IRX7 and other *Arabidopsis* sequences (Kulkarni et al., 2012). The *P. patens* genome also has orthologs of the *GUX* genes, which encode xylan glucoronosyl transferases (Harholt et al., 2012).

Mixed-linkage β-glucans (MLGs) are apparently absent from bryophyte cell walls (Popper and Fry, 2003). The *P. patens* genome lacks members of the *CSLF* and *CSLH* families (Roberts and Bushoven, 2007), which are involved in MLG biosynthesis in cereals (Burton et al., 2006; Doblin et al., 2009; Nemeth et al., 2010; Vega-Sanchez et al., 2012), but absent in dicots. While MLGs have been detected in seedless plants, including *Equisetum* (Sø, 2008) and *Selaginella* (Harholt et al., 2012), there is no evidence that these polymers are synthesized by CSLF and CSLH proteins.

The *P. patens* genome also lacks members of the *CSLB*, *CSLE*, and *CSLG* families found in seed plants. Although the biosynthetic functions of the encoded proteins are unproven, they may be involved in cell wall polysaccharide biosynthesis (Richmond and Somerville, 2000). The *P. patens* genome includes eight *CSLD*s, which have been linked to the biosynthesis of β-1,4 glucan (Park et al., 2011) and mannan (Yin et al., 2011) in angiosperms. The use of *P. patens* as a relatively simple model organism may help to clarify CSLD function.

### PECTINS

Pectin epitopes, including homogalacturonan, β-1,4-galactan, and α-1,5-arabinan were detected by CoMPP and sugar linkage analysis in *P. patens* cell walls (Moller et al., 2007). Desterified pectin and RG-I were also detected by immunofluorescence in leaves and stems of *P. patens* (Kulkarni et al., 2012). Water conducting cells of several species and hyaline cells from *Sphagnum* label preferentially with antibodies directed at the arabinosylated β-1,4-galactan epitope of RG-I (Ligrone et al., 2002; Kremer et al., 2004). The cell walls of some moss species may contain an RG-II-like polysaccharide based on the presence of 2-methyl-fucose and 2-methyl-xylose (apiose and aceric acid were not detected) and release of cross-linked borate by driselase (Matsunaga et al., 2004). However, the polymer was not isolated in sufficient quantities for structural characterization. The *P. patens* genome encodes putative homologs of pectin biosynthesis enzymes AtGAUT1 and the RGXTs, but not AtGAUT7, QUA1, or ARAD1 (Harholt et al., 2012). Putative homologs of CMP-Kdo-synthase have also been identified, further supporting the presence of an RG-II-like polysaccharide in mosses (Matsunaga et al., 2004).

### GLYCOPROTEINS

The presence and functional significance of arabinogalactan proteins (AGPs) in the cell walls of *P. patens* is supported by cross-reactivity with Yariv reagent and monoclonal antibodies, as well as growth inhibition by Yariv reagent and in AGP knockouts (Lee et al., 2005a,b; Moller et al., 2007). *Physcomitrella* AGP glycans contain unusual terminal 3-*O*-methyl-L-rhamnosyl residues in addition to the (1,3,6)-linked galactopyranosyl, terminal arabinofuranosyl and (1,4)-linked glucuronopyranosyl residues typical of angiosperm AGPs (Fu et al., 2007). Genes encoding a classical AGP, three AG peptides, and two fasciculin-like AGPs were identified in a *P. patens* EST database (Lee et al., 2005b) and an AGP was among the proteins identified in a proteomic analysis of dehydration response (Cui et al., 2012). Extensin was weakly detected in *P. patens* by CoMPP. Genome searches identified homologs of GT77 proteins implicated in extensin glycosylation (Harholt et al., 2012), but not extensin itself (Lawton and Saidasan, 2011). However, a comprehensive analysis of cell wall protein genes in *P. patens* has not been reported.

### CALLOSE

Callose has been detected in mosses, including *P. patens*, by aniline blue cytochemistry (Scherp et al., 2001; Tang, 2007; Schuette et al., 2009) and CoMPP (Moller et al., 2007). As in other plants and algae, callose is involved in normal developmental processes, including cytokinesis (Scherp et al., 2001) and spore formation (Schuette et al., 2009), and it also forms in response to wounding (Abel et al., 1989; Scherp et al., 2001; Tang, 2007). The association of callose with different developmental stages and stimuli is not unexpected since the *P. patens* genome contains 12 putative *Callose Synthase* (*CalS*) genes that cluster in three clades with *Arabidopsis* *CalS* genes (Schuette et al., 2009).

### LIGNIN

Most reports of lignin in mosses have not withstood further scrutiny (Weng and Chapple, 2010; Espiñeira et al., 2011). However, the presence of lignin-like compounds in mosses is consistent with identification of *P. patens* homologs of genes encoding lignin biosynthesis enzymes (Xu et al., 2009).

### CUTICLE

Although the protonemal filaments of mosses apparently lack cuticles, a hydrophobic cuticle-like layer has been reported in some moss gametophores and sporophytes (Cook and Graham, 1998; Budke et al., 2011). Although some authors have indicated that *P. patens* gametophores lack a cuticle (Liénard et al., 2008), other histochemical studies have suggested that a cuticle is present (Wyatt et al., 2008) in this species. No analysis of *P. patens* genes potentially involved in cuticle biosynthesis has been reported.

## PROSPECTS

While detailed structures of *P. patens* cell wall polysaccharides are now being revealed, few of the *P. patens* glycosyl transferases have been characterized functionally. Further studies in *P. patens* along with comparative studies that seek to identify cell wall characteristics that correlate with adaptation to diverse habitats can enhance our understanding of cell wall biosynthesis and land plant evolution by elucidating the relationships among glycosyl transferase functional diversification, cell wall structural and biochemical specialization, and the roles of cell wall properties in plant adaptation.

## Conflict of Interest Statement

The authors declare that the research was conducted in the absence of any commercial or financial relationships that could be construed as a potential conflict of interest.
